# Impaired Synaptic Plasticity in a Rodent Model of Intimate Partner Violence‐Related Brain Injury

**DOI:** 10.1002/hipo.70135

**Published:** 2026-09-20

**Authors:** Justin Brand, Eric Eyolfson, Kirsten R. B. Suesser, Emma S. J. Smith, Zoe L. Thompson, Stuart J. McDonald, Jodie R. Gawryluk, Sandy R. Shultz, Brian R. Christie

**Affiliations:** ^1^ Neuroscience Program, School of Medical Sciences, University of Victoria Victoria British Columbia Canada; ^2^ Island Medical Program, University of British Columbia Vancouver British Columbia Canada; ^3^ Department of Neuroscience, School of Translational Medicine Monash University Melbourne Victoria Australia; ^4^ Institute for Aging and Lifelong Health, University of Victoria Victoria British Columbia Canada; ^5^ Department of Psychology University of Victoria Victoria British Columbia Canada; ^6^ Department of Cellular and Physiological Sciences University of British Columbia Vancouver British Columbia Canada; ^7^ Department of Psychology San Diego State University San Diego California USA

**Keywords:** cornu ammonis 1, hippocampus, long‐term potentiation, mild traumatic brain injury, non‐fatal strangulation, polytrauma

## Abstract

Intimate partner violence‐related brain injury (IPV‐BI) predominantly affects women and often reflects both structural trauma from mild traumatic brain injury (mTBI), as well as ischemia/hypoxia from non‐fatal strangulation (NFS). This polytrauma can impair cognitive function, particularly learning and memory processes, which may contribute to the continuation of the cycle of violence. This study investigates the effects of mTBI and hypoxic/ischemic injury on hippocampal long‐term potentiation (LTP), a biological model of learning and memory, to determine if the combination of mTBI+NFS would exacerbate deficits in LTP. Female rats were randomly assigned to either Sham, mTBI, NFS, or mTBI+NFS groups to assess acute injury metrics and LTP capacity 7 days after injury. At the time of injury, NFS and mTBI+NFS groups exhibited significant reductions in blood oxygen saturation, heart rate, and poor neurological assessment metrics. Isolated mTBI and NFS impaired LTP in the CA1 hippocampal region while mTBI+NFS increased synaptic excitability in the CA1 yet impaired CA1 LTP. Contrary to our hypothesis, the combined mTBI+NFS did not exacerbate deficits, but these findings highlight that even a single episode of mTBI or NFS is sufficient to impair hippocampal LTP in the female CA1 region.

## 1 Introduction

Intimate partner violence (IPV) is a worldwide health concern with ~1 in 4 women over the age of 15 years experiencing some form of IPV in their lifetime (Sardinha et al. 2022). IPV often involves physical attacks that target the head and neck which leave survivors at risk of an IPV‐related brain injury (IPV‐BI) (Esopenko et al. 2021). However, IPV‐BI is unique compared to other forms of traumatic brain injury (TBI), such as sports‐related concussion or motor vehicle accidents, because it often involves both mild traumatic brain injury (mTBI; i.e., concussion) and non‐fatal strangulation (NFS) (Breiding et al. 2014; St Ivany and Schminkey 2016; Valera and Berenbaum 2003).

Experiencing an mTBI can cause physical damage to neurons, axons, glial cells and the cerebrovasculature of the brain due to the forces sustained at impact. Following the initial injury, secondary injury mechanisms such as neuroinflammation, oxidative stress, autonomic nervous system dysfunction and neurometabolic abnormalities may exacerbate the physical damage and lead to long‐term consequences (Brand et al. 2023; Giza and Hovda 2014; Pham et al. 2019). NFS results in hypoxia and ischemia by restricting air and blood flow to the brain via blockages of the trachea, carotid arteries, and/or jugular veins and also has the potential for reperfusion injury once blood flow is restored (Strack et al. 2001; Valera et al. 2022). In clinical populations, mTBI has been shown to affect learning and memory (Maas et al. 2017; Mavroudis et al. 2024; Silverberg and Iverson 2011) but evidence of learning and memory deficits related to NFS alone is limited. However, initial clinical and pre‐clinical evidence of mTBI and NFS occurring together suggests deficits are exacerbated in the presence of a concomitant NFS incidence (Bramlett et al. 1999; Davies et al. 2018; Sun, Symons, et al. 2025; Valera et al. 2022). For example, even after controlling for the number of IPV‐related TBIs, women who experience strangulation perform worse on working memory tests (Valera et al. 2022). Similarly, pre‐clinical models of reiterated male‐to‐female violent interactions have shown impaired female hippocampal homeostasis, perturbing the estrogen receptor β/brain derived neurotrophic factor (BDNF) axis, and leading to anxiety‐like behavior and chronic stress (Agrimi et al. 2024) while a concomitant mTBI+NFS injury impaired performance on the Morris water maze beyond that observed following an isolated mTBI or NFS injury (Sun, Symons, et al. 2025) suggesting exacerbated cognitive deficits. Both mTBI and NFS may cause ionic imbalances, mitochondrial toxicity and resulting energy crisis. This overlap may be a mechanistic link to exacerbated cognitive deficits seen by Sun, Symons, et al. (2025) yet this has yet to be explored in the context of learning and memory within the hippocampus.

The hippocampus is a critical brain structure for learning and memory (Anand and Dhikav 2012) and may be involved in the cognitive deficits that IPV‐BI survivors experience as a result of these physical attacks (Baker et al. 2024; Serafim et al. 2024). The leading biological theory of learning and memory is long‐term potentiation (LTP) which represents a strengthening of synapses based on patterns of neuronal activity (Bliss and Collingridge 1993; Eyolfson, Suesser, et al. 2024) and a potential phenomenon that may contribute to symptomatology following IPV‐BI. Few studies have examined the capacity for LTP in both cornu ammonis‐1 (CA1) and dentate gyrus (DG) following mTBI (and none following IPV‐BI), and even fewer have included female animals. This is a significant omission in the field as the CA1 and DG are thought to have specialized functions in learning and memory. The CA1 primarily focuses on encoding spatial memory, novel objects, odors, and fear (Burke et al. 2011; Igarashi et al. 2014; Oliva et al. 2016) while the DG specializes in pattern separation (Neunuebel and Knierim 2014), pattern completion (Nakashiba et al. 2012), and working memory (Sasaki et al. 2018). Given these regions process different aspects of memory, they may display different responses to injury. For example, it has been demonstrated that a single mTBI impairs capacity for LTP in the DG, while there were no impairments in the CA1 (White et al. 2017). However, it is known that the CA1 region demonstrates vulnerability to global ischemia (Anand and Dhikav 2012) which may be of particular importance in the context of NFS and IPV‐BI, yet this remains to be explored.

To date, no research has examined how NFS, either in isolation or in combination with mTBI, influences synaptic plasticity despite its potential relevance to the symptomology of IPV‐BI. IPV‐BI is a complex injury and difficult to replicate in pre‐clinical studies as the complex neurotrauma of mTBI and hypoxic/ischemic injury is often coupled with emotional and interpersonal dimensions (Esopenko et al. 2024). As well, mTBI and hypoxic/ischemic brain injury can occur in settings other than IPV‐BI such as drug overdose and during combat sports (i.e., mixed martial arts). However, our novel models of mTBI, NFS, and their combination allow us to investigate how the common physical injury mechanisms (i.e., mTBI, NFS, or mTBI+NFS) often seen in IPV‐BI affect LTP in female rats. Rats were assigned to Sham, mTBI, NFS, or mTBI+NFS groups, and we hypothesized that rats subjected to mTBI+NFS would display impaired capacity to sustain LTP in both the CA1 and DG hippocampal subregions compared to the mTBI or NFS injury groups.

## 2 Methods

### 2.1 Animals

**FIGURE 1:**
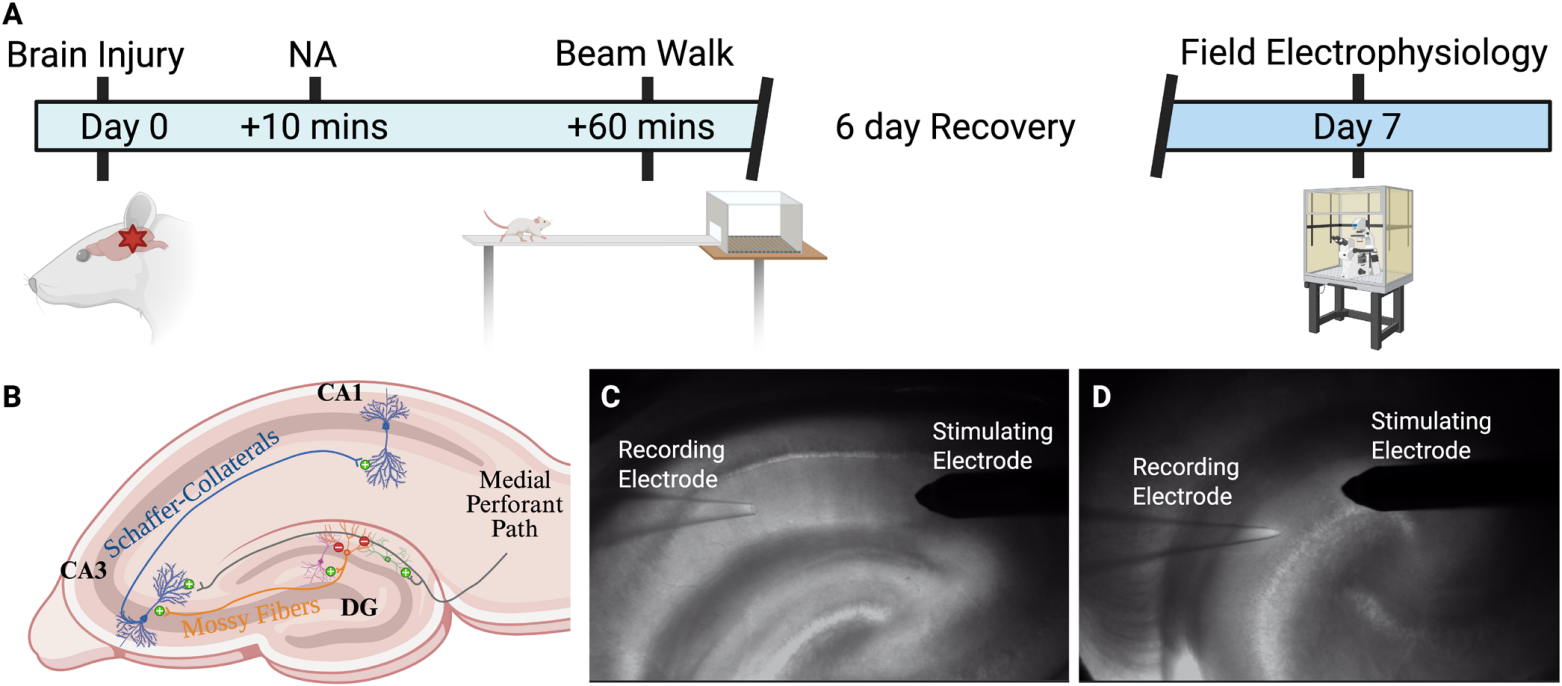
Basic experimental timeline outlining injury timing and acute measures followed by a six‐day recovery before slice preparation for field electrophysiology on post‐injury day seven (A). Schematic of stimulated pathways, Schaffer‐Collaterals for the CA1 and medial perforant path for the DG, adapted with permission from (Eyolfson, Suesser, et al. 2024) (B). Stimulating and recording electrode placements for the CA1 (C) and DG (D). NA, neurological assessment.

Female Sprague–Dawley rats aged 8–9 weeks old were used in this study, as IPV predominantly affects females and often begins in adolescence and early adulthood (Sardinha et al. 2022). Rats were pair‐housed in ventilated cages in a vivarium that was maintained on a 12:12 light–dark cycle, lights off at 7 pm, with *ad libitum* access to food and water. Rats were randomly assigned to Sham (*n* = 12), mTBI (*n* = 12), NFS (*n* = 11), or mTBI+NFS (*n* = 16) and only received a single acute injury. All rats were allowed to recover for 7 days post‐injury before being euthanized for electrophysiological procedures (Figure 1A). All procedures were approved by the University of Victoria Animal Care Committee and performed in compliance with Canadian Council on Animal Care standards.

### 2.2 Brain Injury Models

The mTBI and NFS models were induced as previously described (Sun, Symons, et al. 2025). Briefly, to induce mTBI rats were placed in an anesthetic induction box delivering 5% isoflurane via oxygen carrier gas at 2 L/min for 90 s. Anesthetized animals were then placed in a prone position on a low friction Teflon board within the lateral impact device (Collision Analysis, Calgary, AB, Canada) with the left side of the rat's head placed against an impact plate (30 mm × 13 mm × 3 mm) and in line with the impactor. mTBI was produced by using pneumatic pressure to propel a 50 g weight towards the impact plate through a thrust barrel at speed of 11.4 m/s (Eyolfson, Carr, et al. 2020; Sun et al. 2022). The sham procedure was identical except the impact was not initiated. To induce NFS, rats were placed on a nose cone where 2% isoflurane in oxygen carrier gas was delivered at 1 L/min for the whole procedure. During the first 60 s, rats were given analgesic (0.05 mg/kg Vetergesic Multidose buprenorphine injection; Ceva Animal Health Inc., Cambridge, Canada) subcutaneously to ensure analgesia throughout the NFS procedure. A pulse oximeter (Kent Scientific, CT, USA) was also attached to the hind paw to measure SpO_2_ and HR. The NFS model was then induced for 90 s by placing a silicone band across the trachea that suspended a 1360 g weight attached to the band via carabiner. We measured SpO_2_ and HR at 30‐, 60‐, and 90‐s following NFS induction. After 90‐s, rats had the silicone band/weights and nose cone removed and resuscitated using oxygen and chest massage if spontaneous breathing did not occur after 5 s. Rats were then immediately assessed for acute injury measurements: apnea, pain reflex, and self‐righting reflex. Rats were given a maximum of 10‐min to self‐right and were assigned said value (i.e., 600‐s) if they failed to self‐right. The sham NFS procedure was identical except the silicone band and weight was not suspended across the trachea. Sham animals received the respective sham procedures while isolated injury groups also received the sham procedure of the other injury mechanism (i.e., mTBI received sham NFS, NFS received sham mTBI). For the mTBI+NFS group, animals underwent mTBI or NFS first, followed by the remaining second injury mechanism. There were no differences in injury metrics or electrophysiological properties in the mTBI+NFS group who received either mTBI or NFS first, so these groups were collapsed (see Table S1).

### 2.3 Neurological Assessment

Ten minutes after injury, rats underwent a brief neurological assessment which included four basic neurological outcomes related to simple reflexive and motor tasks to assess impairment following injury. Tasks included a startle response, limb extension, beam walk, and rotating beam tests as previously described (Christie et al. 2019; Meconi et al. 2018). Each task was scored 0–3 (3 indicates perfect score on task) for a total score out of 12. Rats who failed to self‐right 10 min post‐injury were assigned a score of 0 on each task.

### 2.4 Beam Walk

One hour post‐injury, animals were assessed for sensorimotor deficits on the beam walk task as previously described (Eyolfson, Carr, et al. 2020). The home cage was placed at the far end of the beam where animals underwent one training trial on a 100 cm × 4 cm beam followed by four scored trials on a 100 cm × 2 cm beam. Animals were assessed for time to cross the beam (trial time averaged across 4 trials), along with the total number of hind foot slips and falls across all trials. All trials were videotaped and assessed by a researcher blinded to experimental conditions.

### 2.5 Slice Preparation

Seven days following injury, rats were deeply anesthetized before being rapidly decapitated to obtain transverse hippocampal slices as previously described (Christie et al. 2023; Eyolfson, Acosta, et al. 2024). Briefly, the brain was promptly removed and placed in chilled, oxygenated (95% O_2_/5% CO_2_) artificial cerebrospinal fluid (aCSF) containing (in mM) 125 NaCl, 3 KCl, 1.25 NaH_2_PO_4_, 25 NaHCO_3_, 2 CaCl_2_, 1.3 MgCl_2_, 10 Dextrose with an adjusted pH of 7.3 (see Table S2 for list of reagents). Transverse hippocampal slices were sectioned at 400 μm in chilled aCSF using a Compresstome (Precisionary Instruments, USA). Slices were recovered in a holding chamber with continuously oxygenated aCSF at 32°C for 1 h. Slices were then maintained at room temperature until recordings commenced.

### 2.6 Electrophysiology—Field Recordings

Field electrophysiology was performed as previously described (Petersen et al. 2013; White et al. 2017). Briefly, stimulating and recording electrodes were placed ~200 μm apart in either the stratum radiutum of the CA1 (Figure 1C) or the medial perforant path of the DG (Figure 1D). fEPSPs were recorded using glass microelectrodes (0.5–1.5 MΩ) containing regular aCSF and either a concentric or twisted bipolar stimulating electrode. For this experiment, current was adjusted to produce fEPSP ~50% of the maximal fEPSP. A PP was then conducted using an inter‐pulse interval of 50 ms (6×; 15‐s between pairings). To calculate PP ratios, all six PP traces were averaged to create a single trace. The slope of pulse two was divided by slope of pulse one to generate a PP ratio. An input/output (I/O) curve was generated by increasing stimulation magnitude at 15‐s intervals (30–300 μs pulse width). I/O curves assessed peak amplitude of each of the 10 steps as described previously (Kannangara et al. 2015). A 20‐min steady baseline was then recorded before inducing LTP via high‐frequency stimulation (HFS; 50 pulses at 100 Hz, 0.24 ms; 4× with 30‐s intertrain interval). For DG recordings, a γ‐aminobutyric acid A (GABA_A_) antagonist bicuculline methiodide (BMI) (10 μM; Enzo Life Sciences Inc., see Table S2), was perfused over slices throughout the 20‐min baseline and HFS (Eyolfson, Acosta, et al. 2024). Recordings continued in regular aCSF for at least 60‐min post‐conditioning (15‐s interstimulus interval) to quantify the level of LTP induced by the HFS. The first minute after conditioning stimulus is reported as STP (average of first four traces) presented as percent change compared to fEPSP slope of baseline recording. Changes in synaptic plasticity (LTP) were calculated as the average fEPSP slope 55–60 min after conditioning stimulus (average of last 20 traces) compared to 20‐min baseline fEPSP slope and presented as percent change from baseline slope.

### 2.7 Data and Statistical Analysis

Electrophysiological data was recorded using Clampex 11.2.1 (Axon Instruments, CA) and analyzed with Clampfit 10.7 (MDS Analytical Technologies, Toronto, ON, Canada). All statistical analyses were completed using GraphPad Prism 10.1.1 for macOS (La Jolla, CA, USA). SpO_2_ and HR curves were analyzed using a mixed‐design ANOVA with time and injury group as within‐ and between‐group factors, respectively. I/O curves were also analyzed using a mixed‐design ANOVA with pulse intensity (within) and injury group (between) as factors. Normality was assessed using the Shapiro–Wilks Test. Apnea, Pain Reflex, Time to Right, NA, beam walk time to cross, beam walk total number of hind foot slips and falls, PP, STP, and LTP were analyzed using a one‐way ANOVA or Kruskal–Wallis test where appropriate. Presynaptic measures of PP ratio and I/O curves were compared to Sham group only, all other measures included comparisons across groups. For electrophysiological measures, if more than one slice from the same animal was included in the data set, the measures were averaged to generate a single value for that animal and subsequently included in analyses. Electrophysiological measures were not able to be generated for some animals due to exclusion criteria, hence final animal included in the analysis was Sham (*n* = 8), mTBI (*n* = 8), NFS (*n* = 8), and mTBI+NFS (*n* = 12) for the CA1 and Sham (*n* = 8), mTBI (*n* = 10), NFS (*n* = 10), and mTBI+NFS (*n* = 9) for the DG. All data expressed as mean ± standard error of the mean (SEM). Statistical significance was set at *p* < 0.05. Post hoc analysis was performed using Bonferroni or Dunn's multiple comparison corrections where appropriate.

## 3 Results

### 3.1 Immediate Injury Metrics

**FIGURE 2:**
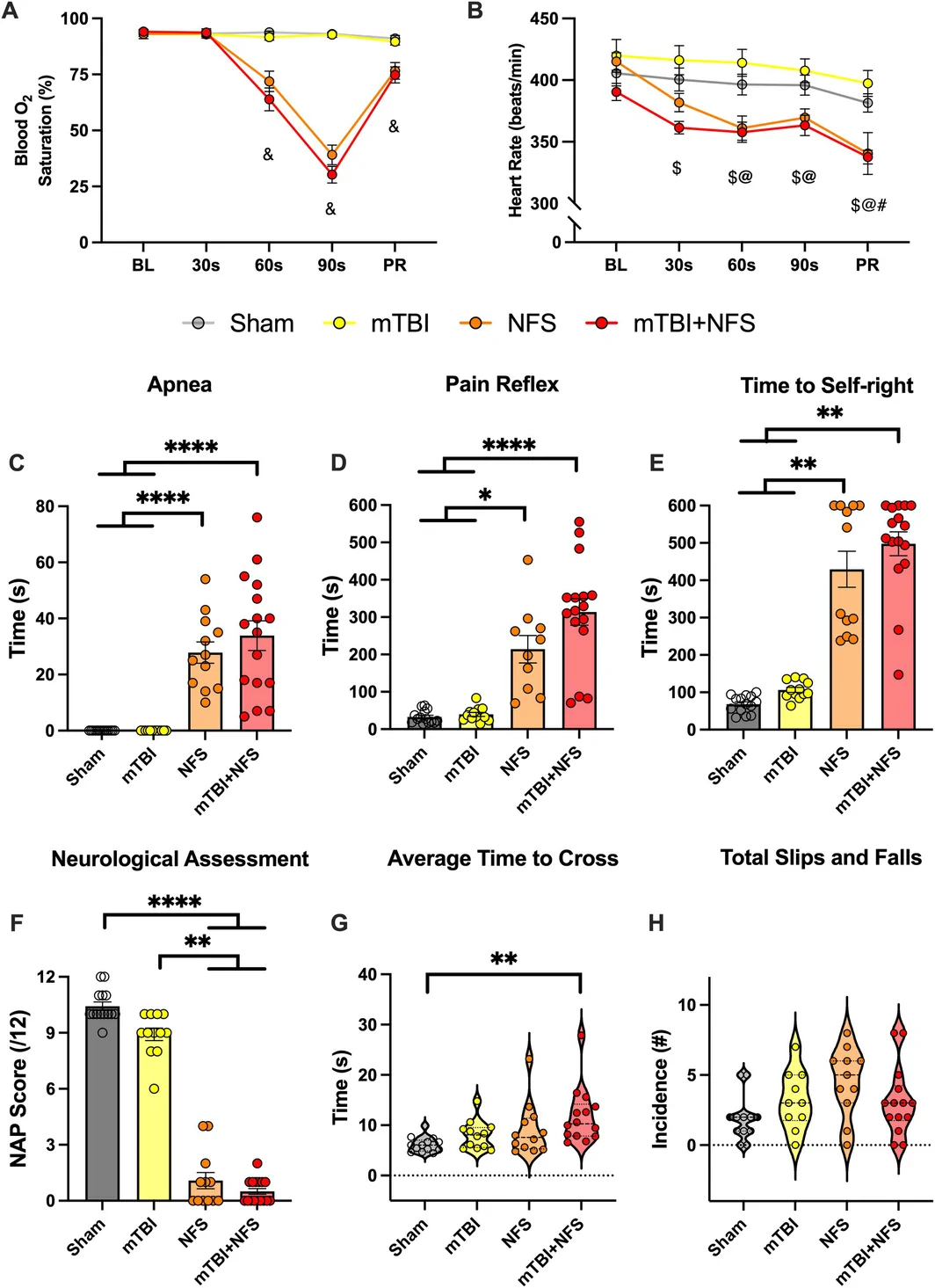
Injury metrics during injury, and at hyperacute time‐points after injury show that injury mechanisms induce impairments among all injury groups. Animals receiving NFS procedure had reduced SpO_2_ (A) and HR (B) at various time‐points throughout injury procedure. Compared to Sham and mTBI, both NFS and mTBI+NFS groups had significantly increased apnea time (C), time to pain reflex (D), and time to self‐right (E). All injury groups had a significantly reduced Neurological Assessment score compared to Sham while NFS and mTBI+NFS also had significantly reduced Neurological Assessment scores compared to mTBI (F). mTBI+NFS had significantly increased average time to cross in the beam walk task (G) but there were differences in the total number of hind foot slips and falls (H). SpO_2_ and HR, mixed‐design ANOVA with time and injury group as factors. All other metrics used Kruskal–Wallis test. Post hoc Bonferroni or Dunn's multiple comparison corrections where appropriate for all graphs. $ indicates mTBI+NFS significantly different from Sham and mTBI, & indicates NFS significantly different from Sham and mTBI, @ indicates mTBI+NFS significantly different than mTBI, # indicates NFS different than mTBI. **p < 0.01, ***p < 0.001, ****p < 0.0001; A–F: data presented as mean ± SEM; F–G: data presented as median and quartiles.

During the NFS or sham NFS procedure, blood oxygen saturation (SpO_2_) and heart rate (HR) were monitored using a pulse oximeter. For SpO_2_, mixed‐design ANOVA revealed there was a significant effect of time (*F*_4,192_ = 105.7, *p* < 0.0001), group (*F*_3,48_ = 57.5, *p* < 0.0001), and time × group interaction (*F*_12,192_ = 37.8, *p* < 0.0001), with the mTBI+NFS and NFS groups having reduced SpO_2_ compared to Sham and mTBI at 60, 90 s, and at time of pain reflex (Figure 2A). For HR, mixed‐design ANOVA revealed a significant effect of time (*F*_4,192_ = 35.7, *p* < 0.0001), group (*F*_3,48_ = 8.05, *p* = 0.0002), and time × group interaction (*F*_12,192_ = 3.37, *p* = 0.0002), with evidence of reduced HR in the mTBI+NFS group at all time points excluding baseline and in the NFS group at 60 and 90 s (Figure 2B). There was a significant effect of group on apnea (*H* = 44.9, *p* < 0.0001, Figure 2C), pain reflex (*H* = 38.97, *p* < 0.0001, Figure 2D), and righting times (*H* = 42.66, *p* < 0.0001, Figure 2E), with the NFS and mTBI+NFS groups having longer times than Sham and mTBI groups (*p* < 0.05 for all measures).

### 3.2 Neurological Assessment Scores of Injury Severity

Kruskal–Wallis test revealed a significant effect of group (*H* = 43.89, *p* < 0.0001, Figure 2F) with NFS and mTBI+NFS groups having significantly decreased neurological assessment scores compared to Sham (*p* < 0.001). Additionally, NFS and mTBI+NFS scores were significantly decreased compared to mTBI alone (*p* < 0.001), suggesting NFS introduces a unique facet to these post‐injury measures.

### 3.3 mTBI+NFS Caused Increased Average Time to Cross in Beam Walk Task

For the beam walk task, the Kruskal–Wallis test revealed that the average time‐to‐cross showed a significant effect of group (*H* = 15.2, *p* = 0.0017). Dunn's post hoc comparisons revealed that compared to Sham, mTBI+NFS had significantly increased average time to cross (*p* = 0.0006, Figure 2G). There were no significant effects for the total number of hind foot slips and falls (*H* = 7.6, *p* = 0.055, Figure 2H).

### 3.4 mTBI+NFS Increases fEPSP Responsiveness Without Altering Presynaptic Neurotransmitter Release

**FIGURE 3:**
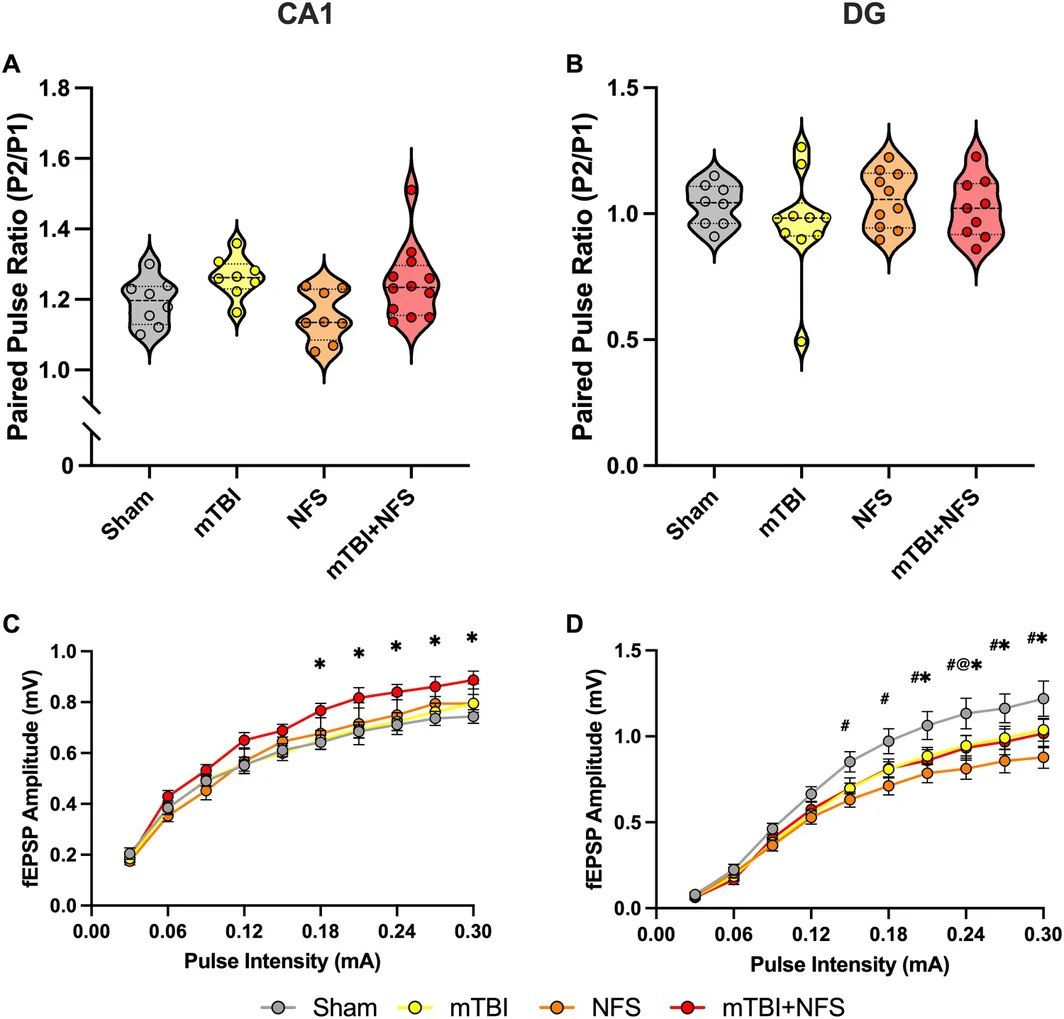
Paired‐pulse ratios were unaltered in both the CA1 and DG but excitation and inhibition imbalances are present in the CA1 and DG. Injury mechanisms did not cause changes in paired‐pulse ratios in the CA1 (A) or DG (B). Compared to Sham, mTBI+NFS caused increased excitation in the CA1 as pulse intensity increased (C), while mTBI, NFS, and mTBI+NFS caused decreased excitation in the DG as pulse intensity increased (D). Paired pulse ratio analyzed with a one‐way ANOVA; I/O curves analyzed with mixed‐design ANOVA with pulse intensity and injury group as factors. Post hoc Bonferroni multiple comparison corrections where appropriate. # mTBI significantly different from Sham, @ NFS significantly different from sham, * mTBI+NFS significantly different compared to sham; A–B: data presented as median and quartiles; C–D: data presented as mean ± SEM.

Paired‐pulse (PP) was used as a measure for changes in presynaptic neurotransmitter release between groups to determine the importance of the type of injury (Jiang et al. 2004; Komaki et al. 2013; White et al. 2017). One‐way ANOVA for PP ratio revealed a significant effect of group (*F*_3,32_ = 3.44, *p* = 0.028) in the CA1; however, no significant differences between groups were found following post hoc comparisons (Figure 3A). In the DG, one‐way ANOVA revealed no significant effects for PP ratio (*F*_3,33_ = 0.79, *p* = 0.051; Figure 3B). Input–output (I/O) curves were built to determine if the injury group affected synaptic transmission and field excitatory postsynaptic potential (fEPSP) responsiveness (Fontaine et al. 2019). Mixed‐design ANOVA revealed significant effects of pulse intensity (*F*_9,288_ = 344.6, *p* < 0.0001) in the CA1 (Figure 3C), post hoc analysis revealed that mTBI+NFS fEPSP amplitude was significantly larger compared to Sham at 0.18 (*p* = 0.045), 0.21 (*p* = 0.029), 0.24 (*p* = 0.038), 0.27 (*p* = 0.046), and 0.3 mA (*p* = 0.015). In the DG, mixed‐design ANOVA revealed significant effects of pulse intensity (*F*_9,297_ = 444.2, *p* < 0.0001), group (*F*_3,33_ = 3.3, *p* = 0.032), and a pulse intensity × group interaction (*F*_27,297_ = 2.5, *p* = 0.0001). Post hoc analysis revealed that compared to Sham, mTBI fEPSP amplitude was significantly decreased at 0.24 mA (*p* = 0.049), NFS fEPSP amplitude was significantly decreased at 0.15 (*p* = 0.015), 0.18 (*p* = 0.00026), 0.21 (*p* = 0.0012), 0.24 (*p* = 0.0001), 0.27 (*p* = 0.0003), and 0.3 mA (*p* < 0.0001); mTBI+NFS fEPSP amplitude was significantly decreased at 0.21 (*p* = 0.033), 0.24 (*p* = 0.038), 0.27 (*p* = 0.045), and 0.3 mA (*p* = 0.034; Figure 3D).

### 3.5 mTBI, NFS, And mTBI+NFS Injuries Reduce LTP in the CA1 Subfield but Not the DG

**FIGURE 4:**
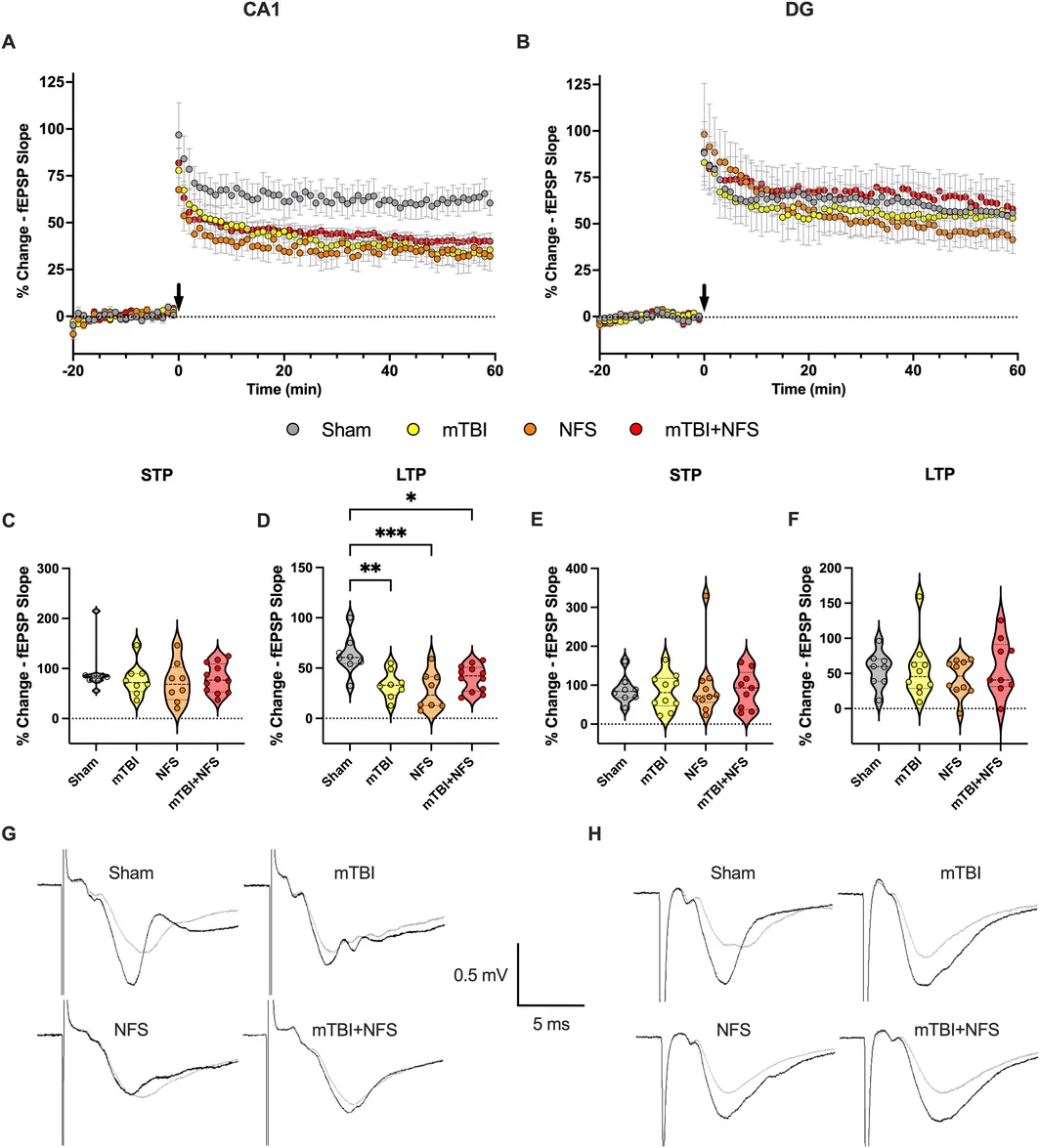
Injury mechanisms caused deficits in LTP in the CA1 only. Average group values for baseline and post‐conditioning for CA1 (A) and DG (B) are displayed to aid in visualization. Within the CA1, there were no differences in STP (C) but mTBI, NFS, and mTBI+NFS showed significant decreases in LTP compared to Sham (D). Within the DG, there were no differences in STP (E) or LTP (F). Average traces for the last five minutes of baseline (gray trace) and the last five minutes of post‐conditioning (i.e., LTP, black trace) for each group are shown to aid in visualizing the changes pre‐ and post‐conditioning for the CA1 (G) and DG (H). STP for both regions and LTP in the DG analyzed using Kruskal–Wallis test with Dunn's multiple comparison corrections where appropriate. CA1 LTP analyzed using one‐way ANOVA with post hoc Bonferroni multiple comparison corrections where appropriate. *p < 0.05, **p < 0.01, ***p < 0.001; A & B data presented as mean ± SEM; C–F presented as median and quartiles.

At 7 days following injury, the capacity for short‐term potentiation (STP; first minute following high‐frequency stimulation) was evaluated, in addition to examining LTP (55–60 min following high‐frequency stimulation) in Schaffer collateral (CA1) and medial perforant (DG) pathways following high‐frequency stimulation (HFS). The average slope of the fEPSP is presented for the pre‐conditioning and post‐conditioning period in Figure 4A for CA1 and 4B for the DG. Kruskal–Wallis test revealed no significant effects on STP in the CA1 (*H* = 1.4, *p* = 0.71, Figure 4C) or DG (*H* = 0.22, *p* = 0.98, Figure 4E). However, one‐way ANOVA for LTP revealed a significant effect of group for the CA1 subfield (*F*_3,32_ = 7.50, *p* = 0.0006). Bonferroni post hoc comparisons revealed that mTBI (*p* = 0.005), NFS (*p* = 0.0006), and mTBI+NFS (*p* = 0.022) displayed significantly reduced LTP compared to Sham, but there were no significant differences between any of the injury groups (Figure 4D). In the DG, Kruskal–Wallis test revealed no significant effects of group for LTP (*H* = 1.46, *p* = 0.69, Figure 4F). Average traces for the final five minutes of baseline (displayed in gray) and last five minutes of post‐conditioning period (i.e., LTP, displayed in black) are presented in Figure 4G for the CA1 and Figure 4H for the DG.

### 3.6 Exploratory Analysis of Relationship Between Acute Injury Metrics and Electrophysiological Measures

Spearman correlation test was used to explore whether any relationships existed between acute injury metrics and electrophysiological measures. This analysis revealed that when data from all groups were pooled, % change EPSP was negatively correlated with apnea (*r* = −0.38, *p* = 0.024) and time to right (*r* = −0.50, *p* = 0.002) in the CA1. However, when investigating the relationships on a group basis, there were no statistically significant relationships. In the DG, there were no statistically significant overall or group level relationships between acute injury metrics and % change EPSP. See Table S3 for full associations between acute injury metrics and electrophysiological measures.

## 4 Discussion

This is the first study to investigate changes in synaptic plasticity following a single acute mTBI, NFS, or both concomitantly. Clinical studies show that physical IPV attacks may increase the likelihood of increased cognitive issues later in life (Baker et al. 2024; Serafim et al. 2024), so we hypothesized that the mTBI+NFS insult would exacerbate deficits in the capacity for LTP when compared to either form of injury alone. Neither mTBI nor NFS alone altered presynaptic neurotransmitter release, but the combination of mTBI+NFS increased excitability in the CA1 region while each injury group displayed decreased excitability in the DG region, as evidenced by the changes in the respective I/O curves. LTP was selectively impaired in the CA1 subfield of the hippocampus in all three injury groups; however, contrary to our hypothesis, all three injury groups exhibited similar degrees of impairment.

Consistent with previous findings (Sun, Symons, et al. 2025), we found that NFS groups (i.e., NFS and mTBI+NFS groups) experienced significant decreases in blood oxygen saturation and heart rate throughout the injury procedure and experienced longer apnea times, time to pain reflex, and time to self‐right, which indicates loss of consciousness and correlate to clinical outcomes (Meconi et al. 2018; Reyes et al. 2022). This may indicate that NFS alone presents as a more severe injury in the hyperacute setting than a mTBI in isolation. Consistent with this notion, the NFS and mTBI+NFS groups also had significantly lower Neurological Assessment scores compared to Sham and mTBI, indicating NFS decreased neurological function after injury but does not exacerbate this deficit in the presence of a concomitant mTBI.

LTP of synaptic plasticity remains the leading biological mechanism for understanding learning and memory processes (Bliss and Collingridge 1993) influenced by presynaptic modifications such as neurotransmitter release, postsynaptic modifications such as increases in receptor numbers or function, extrasynaptic modifications such as reduced neurotransmitter uptake at the synapse, and morphological modifications such as dendritic spine changes (Bliss and Collingridge 1993). Paired‐pulse stimulation can serve as a measure of presynaptic neurotransmitter release (White et al. 2017; Zucker 1989) while I/O curves allow for the investigation into injury effects on synaptic transmission (White et al. 2017), two crucial components that could affect overall synaptic plasticity in the hippocampus. In this study, injury mechanisms did not alter presynaptic PP ratio in the CA1 or DG providing evidence that presynaptic neurotransmitter release does not confound the LTP synaptic plasticity results. However, when using I/O curves to investigate baseline synaptic transmission, at higher pulse intensities, the mTBI+NFS group showed increased excitation compared to sham in the CA1 while mTBI, NFS, and mTBI+NFS groups showed decreased excitation compared to sham in the DG. Specifically for the CA1, mTBI had no effect on I/O curves in the female CA1 (White et al. 2017) but hyperexcitability seen in the mTBI+NFS groups may be explained by the fact that hyperexcitability and seizure activity are apparent when exposing hippocampal slices to hypoxic environments (i.e., 95% N_2_−5% CO_2_ overflow gas) (Kawasaki et al. 1990). As mTBI and episodes of oxygen deprivation can induce an increase in glutamatergic signaling (Giza et al. 2006; Giza and Hovda 2014; Katayama et al. 1990; Kawasaki et al. 1990), mTBI+NFS may be producing a synergistic effect that alters network function increasing the excitability and synaptic transmission of the mTBI+NFS group without altering presynaptic neurotransmitter release. Within the DG, the decrease in baseline synaptic transmission but intact LTP suggests a shift in the balance of excitation and inhibition, potentially increasing inhibition to prevent abnormal neuronal activation (Bao et al. 2011), or a compensatory mechanism unimpeded by injury mechanism which allows for the maintenance of LTP synaptic plasticity.

A significant finding from LTP results is that a single acute episode of mTBI, NFS, or mTBI+NFS can decrease the capacity for LTP in the CA1 region while LTP remains intact in the DG region. Contrary to our hypothesis, mTBI+NFS did not result in greater deficits than an incidence of mTBI or NFS in isolation, potentially reflecting a “floor” effect for reduced synaptic plasticity with the protocol we used. LTP is driven by glutamatergic signaling, activation of *N*‐methyl‐D‐aspartate receptors (NMDAR), and insertion of additional α‐amino‐3‐hydroxy‐5‐methyl‐4‐isoxazole propionate receptors (AMPAR) into the postsynaptic membrane (Eyolfson, Suesser, et al. 2024). Excessive NMDAR activation has been shown to accompany the pathophysiological cascade after TBI, and this may result in pathological levels of calcium influx into activated neurons (Giza et al. 2006). However, given that we did not see any changes in PP ratios in the CA1, the reduced capacity for LTP is likely due to postsynaptic modifications (Schwarzbach et al. 2006) or increased pyramidal neuron cell damage, which is seen particularly in the CA1 region (Anand and Dhikav 2012; Boissard et al. 1992). To our knowledge, this is the first study to focus on synaptic plasticity following hypoxic/ischemic injury via NFS in isolation or in combination with mTBI, which together or separately, are common physical assault mechanisms seen in IPV and other scenarios such as combat sports. Hypoxia alone has been studied in the context of intermittent hypoxia (IH) as a model of obstructive sleep apnea. Results presented here directly follow those of previous studies that found IH impaired LTP in the CA1 but not the DG (Wall et al. 2014). Previous studies also demonstrated that rats exposed to repetitive IH exhibit reductions in population spike amplitudes and LTP in the CA1 (Payne et al. 2004; Xie et al. 2010) and increased latency to find platform times in the water maze task (Goldbart et al. 2003). This suggests that IH impairs spatial learning, a critical function of the CA1 region while the DG is more resistant to a hypoxic/ischemic insult.

This is the first study to characterize the functional alterations in hippocampal synaptic plasticity after both mTBI and concurrent brief hypoxia, but the specific molecular mechanisms involving synaptic proteins, dendrites, neuronal damage, neurotransmitters, and hormones that may underlie functional alterations were beyond the scope of the current study and remain to be investigated. Previous studies have found that key synaptic proteins Synapsin I and postsynaptic density 95 (PSD‐95) are significantly decreased in hippocampal homogenate 48–96 h and 7 days after injury (Ansari et al. 2008; Wakade et al. 2010), while others found increased mean apical and basal dendrite size and decreased expression levels of α‐calcium calmodulin kinase II (α‐CaMKII) in the CA1 (Schwarzbach et al. 2006). Both the hypoxic/ischemic injury of NFS and mTBI trigger excess glutamate release leading to overactivation of NMDAR (Giza and Hovda 2014; Romeu‐Mejia et al. 2019; Wu and Tymianski 2018), and in turn decreases levels of PSD‐95 and other synaptic proteins associated with synaptic transmission (Wang et al. 2023). In the CA1, hypoxic/ischemic injury caused reductions in spine density (Basak et al. 2025; Lippman‐Bell et al. 2021). This imbalance in spine size and density coupled with decreased α‐CaMKII availability is likely to contribute to synaptic plasticity impairments. Neuronal damage may also play a role in synaptic plasticity, with previous studies finding the CA1 showed increased markers for damaged neurons (Hall et al. 2008), increased number of apoptotic cells, and decreased total number of neurons after TBI (Meyer et al. 2012). Similarly, both the CA1 and DG experience significant neuronal loss, while the CA1 sees an increase in microglia (Grady et al. 2003). No studies have specifically looked at NFS and neuronal damage, but one study investigating stroke via middle cerebral artery occlusion for 60 min found that the DG showed an acute increase in neuronal damage markers, but the CA1 became the most severely affected region at 4 days post‐reperfusion (Butler et al. 2002). Compared to TBI alone, CA1 and DG also showed increased neurodegenerative markers 24 h after TBI combined with hypoxia via surgical ventilation (Feng et al. 2012). Similarly, reiterated male‐to‐female violent interactions produced downregulation of estrogen‐mediated signaling, prompting hippocampal neuronal loss and hampered neurogenesis (Agrimi et al. 2024), representing a potential mechanism leading to decreased LTP. As well, investigating catecholamines immediately after injury and prior to endpoint experiments could provide insights into sympathetic responses (Steptoe 1987). Understanding sympathetic responses may provide insights to relevant alterations in baseline excitation.

Here, we report similar findings that just a single instance of NFS or mTBI+NFS is enough to produce functional deficits in CA1 LTP. While important to document, given the deficits seen in repetitive models of mTBI and hypoxia (Allen et al. 2026; Aungst et al. 2014; Hu et al. 2022; Payne et al. 2004; Sun, Allen, et al. 2025; Xie et al. 2010), future work should examine whether these injury mechanisms act synergistically with repeated exposures and incorporate chronic post‐injury timepoints. Our model also investigated an injury sustained at the transition from adolescence to early adulthood when cellular changes in synapse and neuronal number plateau (Juraska and Drzewiecki 2020). However, as IPV is shown to begin in adolescence in humans (i.e., ~15 years old), future work could incorporate earlier injury ages as pre‐clinical models show brain injury sustained in adolescence compared to adulthood exhibit altered microglial function (Eyolfson, Khan, et al. 2020) and distinct patterns of functional deficits including worse spatial memory (Mannix et al. 2017).

In clinical populations, memory deficits are a commonly reported symptom following IPV‐BI (Monahan and O'Leary 1999; Saadi et al. 2022; Smirl et al. 2019), even after controlling for abuse severity, anxious arousal, and post‐traumatic stress disorder (PTSD) symptoms (Valera and Berenbaum 2003). Both mTBI (Snowden et al. 2020; Tremblay et al. 2013) and hypoxic/anoxic injuries (i.e., strangulation) (Peskine et al. 2010; Wright et al. 2017) have been shown to impair memory, and the level of impairment is positively correlated with increasing injury number (Lennon et al. 2023; Smith et al. 2001; Wilbur et al. 2001). Thus, although our presented findings provide novel insights into the consequences of common physical assaults seen in IPV, it is important to note that clinical populations often report multiple instances of IPV‐BI (Valera 2018; Valera and Berenbaum 2003). Future work with a repetitive injury model and extended chronic time‐points (i.e., > 1‐month post‐injury) will encompass post‐injury experiences seen in clinical populations and allow for a conclusion on the long‐term effects of mTBI, NFS, or the combination of the two as is often seen in IPV‐BI.

## 5 Conclusions

In summary, we provide novel functional evidence that a single episode of mTBI, NFS, or the combination of mTBI+NFS can selectively disrupt CA1 plasticity without affecting the DG. Our findings provide evidence that reductions in the capacity for synaptic plasticity may play an important role in the deficits that occur following some of the common physical assault modalities that often accompany IPV.

## Author Contributions

J.B. contributed to Conceptualization, Methodology, Investigation, Formal Analysis, Writing – Original Draft, Writing – Review, Editing, and Visualization. E.E. contributed to Investigation, Formal Analysis, and Writing – Review and Editing. K.R.B.S. contributed to Investigation and Writing – Review and Editing. E.S.J.S. and Z.L.T. contributed to Investigation and Writing – Review and Editing. S.J.M. and J.R.G. contributed to Conceptualization, Supervision, and Writing – Review and Editing. S.R.S. and B.R.C. contributed to Conceptualization, Formal Analysis, Supervision, Funding Acquisition, and Writing – Review and Editing.

## Funding

J.B. was supported by a CGS‐M award from CIHR. E.E. was supported by a MSFHR Fellowship. B.R.C. and S.R.S. were supported by grants from CIHR (FRN 191316) and an NHMRC Ideas Grant (GNT2010763). S.R.S. was supported by a Michael Smith Health Research BC Scholar Award (SCH‐2021‐188). B.R.C. was supported by grants from CIHR (FRN 175042) and NSERC (RGPIN‐2018‐04829).

## Conflicts of Interest

The authors declare no conflicts of interest.

## Supporting information


**Table S1:** Unpaired *t*‐test results for varying injury order in the combination injury group for paired pulse (PP), short‐term potentiation (STP), and long‐term potentiation (LTP) and mixed‐design ANOVA results for input–output (I/O) curves. DF, degrees of freedom.
**Table S2:** List of reagents, supplier, and catalog number for all reagents used.
**Table S3:** Associations between acute injury measures (apnea, pain reflex, time to right, and Neurological Assessment score) and electrophysiological measures in the CA1 and DG. % change EPSP represents the % LTP (55–60 min following high‐frequency stimulus), PPR; paired‐pulse ratio, I/O Max; Input/Output max at a pulse intensity of 0.3 mA. Overall group is all the individual groups data pooled. Relationships between Apnea and Sham and mTBI groups could not be calculated as all Sham and mTBI groups had an apnea score of 0 making the relationship a vertical line. Red, bolded text indicates a significant relationship where *p* < 0.05.

## Data Availability

The data that support the findings of this study are available from the corresponding author upon reasonable request.
