# Supplementary material for: Impaired Synaptic Plasticity in a Rodent Model of Intimate Partner Violence‐Related Brain Injury
Source: Hippocampus. 2026 Sep 20;36(5):e70135. doi: 10.1002/hipo.70135 (PMC13590904; doi:10.1002/hipo.70135)
Supplement: Supplementary file 1 [file HIPO-36-0-s001.docx]

| Unpaired t-test | Mean mTBI+NFS | Mean NFS+mTBI | | t-statistic | DF | | p-value |
| --- | --- | --- | --- | --- | --- | --- | --- |
| PP | 1.287 | 1.18 | | 1.99 | 10 | | 0.08 |
| STP | 83.09 | 74.43 | | 0.48 | 10 | | 0.65 |
| LTP | 42.62 | 35.42 | | 0.92 | 10 | | 0.38 |
|  | | | | | | | |
| I/O EPSP | Mixed-Design ANOVA Table | | | | | | |
| Factor | Pulse Intensity | | Group | | | Pulse Intensity x Group | |
| F(p-value) | 148.1 (p<0.0001) | | 0.01 (p=0.92) | | | 1.19 (p=0.31) | |

**Supplementary Table 1.** Unpaired t-test results for varying injury order in the combination injury group for paired pulse (PP), short-term potentiation (STP), and long-term potentiation (LTP) and mixed-design ANOVA results for input-output (I/O) curves. DF = degrees of freedom.

| **Reagent** | **Supplier** | **Catalog Number** |
| --- | --- | --- |
| Sodium Chloride (NaCl) | Fischer Scientific | S271-3 |
| Potassium Chloride (KCl) | Fischer Scientific | P217-500 |
| Sodium Phosphate Monobasic (NaH_2_PO_4_) | Fischer Scientific | S369-500 |
| Sodium Bicarbonate (NaHCO_3_) | Fischer Scientific | S233-500 |
| Calcium Chloride Dihydrate (CaCl_2_ • 2H_2_O) | Fischer Scientific | C79-500 |
| Magnesium Chloride Hexahydrate (MgCl_2_ • 6H_2_O) | Fischer Scientific | M33-500 |
| Dextrose (D-Glucose) Anhydrous (C_6_H_12_O_6_) | Fischer Scientific | D16-3 |
| Agarose (C_12_H_18_O_9_) | Fischer Scientific | BP160-500 |
| Bicuculline methiodide | Enzo Life Sciences Inc | BML-EA149-0059 |
| Vetergesic Multidose Buprenorphine Injection | Ceva Animal Health Inc | (01)03411112937103 |

**Supplementary Table 2.** List of reagents, supplier, and catalog number for all reagents used.

| % change EPSP (Spearman r, p-value) | | | | | | |
| --- | --- | --- | --- | --- | --- | --- |
|  |  | Overall | Sham | mTBI | NFS | mTBI+NFS |
| CA1 | Apnea | **-0.38, 0.024** |  |  | -0.31, 0.46 | -0.50, 0.10 |
|  | Pain Reflex | -0.25, 0.15 | -0.44, 0.28 | -0.05, 0.94 | 0.49, 0.36 | -0.27, 0.39 |
|  | Time to right | **-0.50, 0.002** | -0.43, 0.30 | -0.58, 0.14 | -0.56, 0.15 | -0.35, 0.26 |
|  | NA score | 0.33, 0.051 | -0.04, 0.95 | -0.08, 0.86 | 0.0, >0.99 | -0.14, 0.65 |
|  |  |  |  |  |  |  |
| DG | Apnea | -0.05, 0.77 |  |  | 0.18, 0.63 | 0.16, 0.66 |
|  | Pain Reflex | 0.02, 0.90 | 0.04, 0.96 | 0.61, 0.07 | 0.60, 0.13 | 0.18, 0.63 |
|  | Time to right | 0.13, 0.44 | -0.04, 0.96 | 0.16, 0.66 | 0.0, >0.99 | 0.06, 0.89 |
|  | NA score | 0.17, 0.31 | 0.31, 0.57 | -0.39, 0.27 | 0.19, 0.60 | 0.22, 0.54 |
| PPR (Spearman r, p-value) | | | | | | |
|  |  | Overall | Sham | mTBI | NFS | mTBI+NFS |
| CA1 | Apnea | -0.03, 0.88 |  |  | -0.38, 0.36 | **0.61, 0.04** |
|  | Pain Reflex | -0.02, 0.93 | -0.06, 0.90 | 0.14, 0.75 | 0.26, 0.66 | -0.23, 0.47 |
|  | Time to right | -0.02, 0.93 | 0.07, 0.88 | 0.59, 0.13 | -0.47, 0.25 | 0.38, 0.22 |
|  | NA score | 0.19, 0.27 | 0.50, 0.21 | 0.55, 0.16 | 0.39, 0.34 | 0.06, 0.86 |
|  |  |  |  |  |  |  |
| DG | Apnea | 0.25, 0.14 |  |  | 0.09, 0.81 | **0.88, 0.003** |
|  | Pain Reflex | 0.001, 0.99 | **-0.83, 0.015** | -0.29, 0.43 | -0.33, 0.43 | 0.55, 0.13 |
|  | Time to right | 0.05, 0.76 | **-0.92, 0.002** | 0.21, 0.55 | -0.21, 0.56 | 0.45, 0.22 |
|  | NA score | 0.04, 0.82 | 0.25, 0.64 | 0.23, 0.52 | 0.28, 0.43 | 0.36, 0.35 |
| I/O Max (Spearman r, p-value) | | | | | | |
|  |  | Overall | Sham | mTBI | NFS | mTBI+NFS |
| CA1 | Apnea | 0.12, 0.47 |  |  | -0.45, 0.26 | -0.41, 0.19 |
|  | Pain Reflex | 0.31, 0.07 | -0.08, 0.85 | 0.19, 0.67 | -0.20, 0.71 | -0.20, 0.52 |
|  | Time to right | 0.21, 0.23 | -0.14, 0.75 | -0.20, 0.63 | -0.35, 0.40 | 0.03, 0.92 |
|  | NA score | **-0.35, 0.035** | 0.24, 0.57 | -0.22, 0.59 | 0.0, >0.99 | **-0.73, 0.01** |
|  |  |  |  |  |  |  |
| DG | Apnea | **-0.43, 0.009** |  |  | -0.07, 0.87 | -0.62, 0.09 |
|  | Pain Reflex | -0.30, 0.08 | 0.48, 0.24 | 0.08, 0.84 | **-0.76, 0.037** | -0.17, 0.68 |
|  | Time to right | **-0.46, 0.005** | 0.24, 0.58 | 0.0, >0.99 | -0.08, 0.83 | -0.68, 0.052 |
|  | NA score | **0.34, 0.04** | 0.12, 0.86 | **-0.68, 0.035** | 0.33, 0.35 | -0.44, 0.25 |

**Supplementary Table 3.** Associations between acute injury measures (apnea, pain reflex, time to right, and Neurological Assessment score) and electrophysiological measures in the CA1 and DG. % change EPSP represents the % LTP (55-60 mins following high-frequency stimulus), PPR; paire-pulse ratio, I/O Max; Input/Output max at a pulse intensity of 0.3 mA. Overall group is all the individual groups data pooled. Relationships between Apnea and Sham and mTBI groups could not be calculated as all Sham and mTBI groups had an apnea score of 0 making the relationship a vertical line. Red, bolded text indicates a significant relationship where p<0.05.
